# Association of Self-Efficacy and Outcome Expectations with Physical Activity in Adults with Arthritis

**DOI:** 10.1155/2013/621396

**Published:** 2013-10-24

**Authors:** Thelma J. Mielenz, Kathryn L. Kubiak-Rizzone, Kimberly J. Alvarez, Patrick R. Hlavacek, Janet K. Freburger, Carol Giuliani, Vicki S. Mercer, Leigh F. Callahan

**Affiliations:** ^1^Department of Epidemiology, Mailman School of Public Health, Columbia University, 722 West 168th Street, Room 512, New York, NY 10032, USA; ^2^Division of Physical Therapy, University of North Carolina at Chapel Hill, 3030 Bondurant Hall, CB No. 7135, Chapel Hill, NC 27599-7135, USA; ^3^Cecil G Sheps Center for Health Services Research, University of North Carolina at Chapel Hill, CB No. 7590, 725 Martin Luther King, Jr. Boulevard, Chapel Hill, NC 27599-7590, USA; ^4^Thurston Arthritis Research Center, University of North Carolina at Chapel Hill, 3300 Thurston Building, CB No. 7280, Chapel Hill, NC 27599-7280, USA

## Abstract

*Background and Purpose*. The purpose of this study is to determine whether higher baseline levels of (a) self-efficacy for physical activity, (b) self-efficacy for arthritis self-management, and (c) outcome expectations for exercise are associated with higher physical activity levels following an exercise intervention for adults with arthritis. *Methods*. A secondary analysis of the intervention cohort (*n* = 130) within a randomized controlled trial of the People with Arthritis Can Exercise program was performed. Multiple linear regression evaluated the relationship between physical activity at a time point three months after the completion of an exercise intervention and three main explanatory variables. *Results*. After controlling for baseline physical activity, neither self-efficacy for arthritis self-management nor outcome expectations for exercise related to three-month physical activity levels. There was a relationship between three-month physical activity and self-efficacy for physical activity. *Conclusions*. Future research is needed to evaluate the ability of self-efficacy-enhancing programs to increase physical activity in adults with arthritis.

## 1. Introduction

The benefits of physical activity are numerous and range from disease prevention and management to the enhancement of mental and physical well-being. In adults of all ages, physical activity is associated with lower death rates; prevention and management of hypertension; maintenance of strength, agility, and bone mass; and reduction in the symptoms of certain mental illnesses such as depression and anxiety [1]. Among those with chronic disabling conditions like arthritis, physical activity has additional benefits. For example, aerobic and resistive exercise programs have been shown to significantly decrease pain and self-reported disability and improve strength, maximum walking speed, maximal aerobic capacity, and functional performance measures [2–5].

On the other hand, inactivity among adults with arthritis is associated with several health consequences, including pain, onset of disability, loss of independence in activities of daily living, and decreased quality of life [6, 7]. Physically inactive adults with arthritis also incur substantially higher medical costs compared to more active individuals with arthritis. In one year, the medical costs associated with inactivity among adults with arthritis have been shown to exceed $1200 per person [8]. 

To achieve health benefits and avoid the consequences of inactivity, the Surgeon General in 1996 recommended that all Americans accumulate at least 30 minutes of moderate intensity physical activity on most days of the week [9]. This recommendation remains current as one objective of Healthy People 2020 is to “increase the proportion of adults who engage in aerobic physical activity of at least moderate intensity for at least 150 minutes/week” [10]. This is equivalent to 5 sessions of physical activity per week at 30 minutes in length. Despite these guidelines, however, most adults do not lead physically active lifestyles. Data from the 2007 Behavioral Risk Factor Surveillance System (BRFSS) indicate that only 40% of adults with arthritis engage in regular leisure-time physical activity sufficient to meet the recommendations, a small increase from 38.3% in 2001 [11, 12]. The high prevalence of inactivity among adults with arthritis is a problem that must be addressed.

Physical activity and exercise are often used interchangeably, but there are key distinctions between the two terms. Whereas exercise is planned or structured, physical activity may be accumulated throughout the day by making lifestyle changes such as taking the stairs instead of the elevator or parking farther away from a store entrance to increase walking distance. As Kohl et al. [13] noted, “this lifestyle approach to activity intervention may be more appealing than more structured methods to those who are most sedentary and unfit and therefore are at greatest risk” (page 275). Additionally, it may be easier to sustain increases in lifestyle physical activity levels than to continue participation in structured exercise programs, as approximately 50% of structured exercise program participants drop out within six months to a year [14]. Considering these potential benefits, physical activity is the outcome of interest in the present study.

Numerous psychosocial factors have also been proposed to relate physical activity behavior and, given their modifiability, are potential areas of interventions to help increase physical activity levels. Self-efficacy and outcome expectations, central constructs of the Social Cognitive Theory by Bandura [15–17], are two important psychosocial determinants of physical activity behavior. Self-efficacy, or confidence in one's ability to perform a given behavior despite obstacles, is a task-specific construct that influences the amount of time and effort individuals are willing to invest in order to overcome barriers [15–17]. Outcome expectations, the anticipated consequences of a given behavior, influence behavior by serving as incentives (positive outcomes) or disincentives (negative outcomes) [15–17]. Self-efficacy has received the most consistent support of any psychosocial factor as a strong determinant of physical activity behavior [18–22]. Despite receiving less attention than self-efficacy in the literature, there is strong support for the relationship between outcome expectations and physical activity levels [23–28].

Through this study, we attempted to increase current understanding of factors, specifically self-efficacy and outcomes expectations, associated with physical activity behavior among adults with arthritis. The primary objectives were to determine whether higher baseline levels of (a) self-efficacy for physical activity, (b) self-efficacy for arthritis self-management, and (c) outcome expectations for exercise are associated with higher physical activity levels following the cessation of a six-week structured exercise intervention. We hypothesized that higher baseline levels of all three measures would be associated with higher physical activity levels three months after completion of an exercise intervention.

## 2. Methods 

### 2.1. Study Design

This was a secondary analysis of data collected from a randomized controlled trial (RCT) evaluating the effectiveness of the People with Arthritis Can Exercise (PACE) program on key arthritis-related health outcomes [29]. The primary RCT compared intervention group members participating in the PACE program with a delayed control group. The Medical Institutional Review Board at University of North Carolina at Chapel Hill approved the study, and all participants gave informed consent to participate. 

Sedentary adults with arthritis (*n* = 346) were recruited from 18 sites across the state of North Carolina by PACE instructors and the investigators' community contacts (e.g., family practice offices) and local advertisements. The sites were distributed throughout the state in urban and rural counties with varying percentages of African American residents to reflect the state's diversity. Adults 18 years of age or older with self-reported arthritis were eligible to participate if they experienced any limitations in their daily activities as a result of arthritis or joint symptoms. Individuals using wheelchairs were allowed to participate if they could independently transfer to and from a straight-backed chair and toilet. Individuals were excluded if they had any physical or psychological conditions precluding their participation in exercise activities. Individuals who reported exercising regularly on three or more days per week for 20 minutes or more each day were excluded in an effort to target sedentary individuals.

Following completion of the baseline assessment, participants were randomly assigned to either the intervention or control group. Intervention group members were enrolled in PACE classes beginning within the next week, while controls were offered the PACE intervention after completion of the initial 8-week intervention.

The basic PACE program, designed by the National Arthritis Foundation, consists of gentle strengthening, balance, range-of-motion, and endurance exercises at a level appropriate for individuals with functional limitations plus education in proper body mechanics, relaxation techniques, and behavioral strategies to build self-esteem. PACE was modified slightly and renamed the Arthritis Foundation Exercise Program in 2005. Arthritis Foundation-trained instructors led one-hour classes two days per week for eight weeks at the 18 statewide community centers.

### 2.2. Study Sample

This study examined baseline and three-month data from the intervention group only (*n* = 171). At baseline and three months following the intervention, participants completed a battery of self-report, written questionnaires that included the Self-Efficacy for Physical Activity (SEPA) scale, the Rheumatoid Arthritis Self-Efficacy (RASE) scale, the Outcome Expectations for Exercise (OEE) scale, and the Physical Activity Scale for the Elderly (PASE). Participants completed baseline questionnaires in person. Three-month follow-up questionnaires were mailed to the participants. Telephone interviews were conducted with those subjects who failed to return the follow-up questionnaires. Due to variation in the timing of mailings and telephone interviews, the three-month questionnaires were completed anywhere from 2.75 to 4 months following the baseline assessment. The sample for this study included intervention group participants who attended at least one PACE class and who completed the three-month follow-up questionnaires (*n* = 130).

### 2.3. Measures

#### 2.3.1. Self-Efficacy for Physical Activity Scale

The SEPA scale is a five-item scale with five-point Likert response levels assessing respondents' confidence in their ability to be physically active despite common barriers (adverse weather, lack of time, when tired, in a bad mood, or on vacation). A summary score is calculated by averaging responses to all five items, yielding a possible range of one to five points. Higher scores reflect higher levels of self-efficacy for physical activity [30].

The SEPA has shown strong internal consistency across multiple studies (*r* = 0.76–0.85) and significantly differentiates individuals in different stages of exercise behavior change [30, 31]. Two-week test-retest reliability is 0.90 [30]. Construct validity has been supported through associations with numerous measures of physical activity [26, 28, 32–34].

#### 2.3.2. Rheumatoid Arthritis Self-Efficacy Scale

The RASE is a 28-item scale with a five-point Likert response format that measures respondents' self-efficacy for arthritis self-management behaviors. Although developed for individuals with rheumatoid arthritis, the test is applicable to individuals with all forms of arthritis. There are three questions that deal specifically with physical activity. Total score is computed by summing the responses, yielding a possible range of 28 to 140 points. Higher scores reflect higher self-efficacy for arthritis self-management. 

Construct validity of the RASE has been supported by correlation with the Arthritis Self-Efficacy Scale's “other” subscale (*r* = 0.31) and an association between changes in the RASE and changes in the Arthritis Self-Efficacy Scale's “pain” and “other” subscales (*r* = 0.35 and 0.32, resp.). The RASE has also demonstrated sensitivity to change following a self-management education program. One- and four-week test-retest reliability coefficients are 0.69 and 0.89, respectively [35, 36].

#### 2.3.3. Outcome Expectations for Exercise Scale

The OEE scale consists of nine items with five-point Likert response levels assessing respondents' agreement with potential benefits of physical activity for older adults. A summary score is calculated by averaging responses to all five items, yielding a possible range of one to five points. Higher scores reflect higher outcome expectations for exercise. Internal consistency alpha coefficients and two-week test-retest reliability are 0.89–0.93 and 0.76, respectively. Criterion and construct validity have been supported by significant associations with exercise behavior and self-efficacy [27, 37, 38]. One item “exercise gives me a sense of personal accomplishment” was inadvertently omitted from the scale employed in this study.

#### 2.3.4. Physical Activity Scale for the Elderly

The PASE assesses self-reported participation in physical activities of varying intensities over the past seven days. Total score is computed using a formula in which each activity is weighted to reflect energy expenditure. PASE scores may range from 0 to 400 or greater, with higher scores indicating higher levels of physical activity. Validity has been supported by significant correlations between PASE and numerous measures of health and function in populations with and without arthritis. The three- to seven-week test-retest reliability coefficient is 0.75 [39–42].

## 3. Data Analysis

All analyses were conducted using SPSS Version 13.0 and STATA Versions 8.0 and 9.0. Of the 130 participants who completed 3-month follow-up surveys, seven individuals (5.4%) were missing 3-month PASE scores, 21 participants (16.2%) were missing baseline PASE scores, and less than 5% were missing data on the other variables. Participants with missing data were excluded from multivariate analyses. 

Multiple linear regression analyses and more specifically analysis of covariance (ANCOVA) were performed to determine whether higher baseline levels of (a) self-efficacy for physical activity (SEPA), (b) self-efficacy for arthritis self-management (RASE), and (c) outcome expectations for exercise (OEE) are associated with higher physical activity levels (PASE) following the cessation of a structured exercise intervention for adults with arthritis. Because both the RASE and the OEE measures had a nonlinear relationship with the PASE, these data were dichotomized. As no established cut-points are available for these scales and in the absence of other logical cut-points, categories were differentiated using a mean or median split based upon the distribution of the variable. The RASE scale displayed a fairly normal distribution and, thus, was dichotomized using the mean (105.8). With a negatively skewed distribution, the OEE scale was dichotomized using a median cut-point (4.00). 

 Eight covariates (age, race, gender, education, functional limitation, pain, stiffness, and fatigue) were evaluated for their roles as potential confounders. These eight demographic and health-related factors were chosen because they have been shown to significantly affect physical activity behavior in the adult and older adult populations [20, 21, 43, 44]. Functional limitation was measured via the Health Assessment Questionnaire (HAQ) [45]. Pain, stiffness, and fatigue were measured with 10 cm visual analog scales. 

Three separate multivariate analyses were conducted to evaluate the relationship between each of the three main explanatory variables (SEPA, RASE, and OEE) and the 3-month PASE. The analyses were conducted on the three complete models, consisting of the main explanatory variable, the eight covariates, and the baseline PASE. Partial *F*-tests with nine degrees of freedom to test all of the interaction terms together were not significant in any of the models. As a result, no interaction terms were included. Covariates were eliminated from the model one at a time by the change-in-estimate method to evaluate the relative change in the estimated effect on 3-month PASE for the main explanatory variable [46]. The order of elimination for each model was unique and determined from the results of the bivariate analyses. All covariates were included in the initial model because covariates can behave unexpectedly when adjustment for other variables is taken into account. After the final models were determined, the adjusted mean values of 3-month PASE were calculated for the 4 levels of SEPA and for the 2 levels of the RASE and OEE. 

## 4. Results

Of the 175 participants in the intervention group, 130 completed 3-month surveys with a response rate of 74.3%. Forty-five individuals from the original cohort were lost to follow-up. Attrition over the three months was due to study withdrawal, illness, invalid contact information, and unknown reasons. 

 Demographic characteristics are highlighted in Table 1. Research participants (*n* = 130) were predominantly female (85%) and Caucasian (75%), with a mean age of 72 years. More than half of the participants (59%) possessed higher than a high-school level education. They exhibited high OEE (4.1, range = 1–5). As might be expected from community dwelling adults, participants reported minimal functional impairment based on HAQ scores (mean = 0.99, range = 0–2.5). Baseline levels of all other variables were moderate as illustrated in Table 2.

The unadjusted relationship between 3-month PASE and SEPA was moderate but significant (data not shown: *r* = 0.30, *P* < 0.01). The unadjusted mean differences in 3-month PASE between the dichotomized levels of RASE and OEE were minimal and failed to reach significance (data not shown: 8.1, *P* = 0.42, and 10.8, *P* = 0.28, resp.). None of the eight covariates in the conceptual model were confounders. Elimination of each variable from the model resulted in a negligible change in the adjusted mean value of the outcome for each main explanatory variable. The only variable included in the three final models was baseline PASE to control for baseline differences in physical activity levels. 

Table 3 presents mean 3-month PASE values for the three main explanatory variables after adjustment for baseline PASE. The average adjusted 3-month PASE score for individuals with high RASE was 13.9 points higher than those with low RASE. The adjusted mean difference in 3-month PASE values between individuals with high and low OEE was 8.8 points. With each one-point increase in SEPA, mean adjusted 3-month PASE scores were 14.5 points higher. The relationship between SEPA and 3-month PASE scores was statistically significant. 

To interpret the clinical relevance of these results, it is helpful to understand the formula used to calculate a PASE summary score. A weighted value is attributed to each physical activity task in the scale based upon the estimated energy requirements of the task. This weighted value is multiplied by the approximate number of hours per day spent in performance of the task. The resulting products for each task are then added together to obtain a total physical activity score [41]. 

After controlling for baseline PASE, every 1-point increase in SEPA equated to a 14.5-point increase in adjusted mean 3-month PASE score. The 29.0-point difference in adjusted mean physical activity between individuals with a SEPA score of 4.0 versus 2.0 may correspond to an additional six hours per week of moderate or strenuous recreational activities (weighted value of 23 ∗ frequency of 1.29 = 29.7 points), or an additional eight hours per week of walking outside home (weighted value of 20 ∗ frequency of 1.5 = 30 points). 

## 5. Discussion

Results of the present study indicate that higher baseline levels of self-efficacy for physical activity are associated with higher physical activity levels three months after the cessation of a structured exercise intervention for adults with arthritis. These findings provide support for one of the three a priori hypotheses. The other two hypotheses that baseline self-efficacy for arthritis self-management and outcome expectations for exercise would also relate to 3-month PASE scores were not supported. Although individuals with higher RASE and OEE at baseline were more physically active at three months than those with low RASE and OEE, the differences were not statistically significant. 

The Surgeon General recommends that adults accumulate at least 150 minutes of moderate intensity physical activity over the course of a week in order to experience benefits. Considering this recommendation, our results suggest that high self-efficacy for physical activity may yield important health benefits for adults with arthritis [1, 10].

The results are somewhat consistent with findings from studies of healthy adults. The relationship between self-efficacy and physical activity is significant across populations. In the general adult population, however, outcome expectations for exercise have also been shown to relate to physical activity levels [47]. Although this relationship exhibited a trend in the expected direction, it was insignificant in the present study of adults with arthritis. 

One possible explanation for the lack of association between OEE and 3-month PASE is the negatively skewed distribution of the OEE scale. More than 88% of participants reported outcome expectations greater than or equal to 3.5, and 66% reported values of 4.0 or greater. This prevalence of high values suggests a possible ceiling effect of the 1–5 scale that may have masked any potential influence of outcome expectations on physical activity. The omission of one item from the OEE scale may also have contributed to the insignificant results. Finally, statistical power was lost by dichotomizing this variable.

Alternatively, perhaps the results reflect a true difference between healthy adults and those with arthritis. Adults with limitations in daily activities due to joint symptoms may be aware of the many potential benefits of exercise and physical activity, but because of their pain and physical limitations, they may not believe that the benefits are applicable to them. Outcome expectations may not relate to their physical activity behavior if they do not expect to personally experience the benefits of physical activity.

Although the relationship between RASE and 3-month PASE failed to reach statistical significance, the 13.9 point difference in adjusted mean physical activity between individuals with high and low levels of RASE is noteworthy. Such a difference may correspond to an additional four hours per week of walking outside the home (weighted value of 20 ∗ frequency of 0.64 = 12.8 points) or an additional three hours per week of muscle strengthening exercises (weighted value of 30 ∗ frequency of 0.43 = 12.9 points). 

An explanation for the nonsignificant relationship between RASE and 3-month PASE is that the self-efficacy was not specific to physical activity. Self-efficacy is defined as confidence in one's ability to perform a given task [15–17, 48]. In the present study, the task of interest was physical activity, not arthritis self-management. Perhaps the results illustrate the task-specific nature of self-efficacy. It is also possible that due to the dichotomization of the RASE scale, this study simply was not powerful enough to detect a statistically significant difference in 3-month PASE.

### 5.1. Limitations

Intervention participants from the primary RCT were treated as a cohort for this secondary analysis. Although it was the intent for every individual to be exposed to the same intervention, it is possible that the PACE program varied or some participants could have been differentially affected by the PACE program. As a result, the intervention could have mediated the relationship between self-efficacy and physical activity. The primary outcome measures of the randomized trial were symptoms (pain, stiffness, and fatigue), self-report, and performance-based measures of physical function and self-report physical activity assessed by the PASE [29]. Improvements in symptom outcomes and performance-based upper and lower extremity function were found, but no significant improvements in physical activity were found [29]. As mentioned above, the PACE intervention itself may bias the interpretation of the results.

The self-report nature of the PASE may have presented a biased representation of participants' true physical activity levels, and the scoring algorithm may have overestimated the time spent in performance of household tasks. The validity of the PASE has been repeatedly supported and we believe that the scale provided an adequate reflection of actual physical activity behavior [39, 40, 42]. However, limitations of PASE are in the literature, including (1) lower validity for women, (2) floor effects, and (3) physical activity bias towards colder climates (e.g., ice skating); all of these limitations could bias participants' true physical activity levels [39, 42, 49, 50]. Another limitation of PASE in our study was the relatively high number of missing data (16.1%).

### 5.2. Strengths

 As a result of the broad definition of arthritis used in this study, results are not limited to those with a specific type of arthritis, but, rather, are applicable to a larger population of adults with limitations in daily activities due to joint symptoms. In addition, over 25% of the participants were non-Caucasian, and more than one third possessed a high-school education or less. The participants in most existing studies of physical activity determinants are healthy, well-educated adults, and at least 95% are Caucasian [23, 25, 51–53]. The greater diversity of participants in this study suggests that the results are applicable to a wider range of individuals.

 The use of two self-efficacy scales was another strength of the study. Evaluating the relationship between 3-month PASE and scores on both the SEPA and RASE provided a more thorough assessment of the impact of self-efficacy on physical activity among adults with arthritis. 

### 5.3. Implications

 Important implications for clinical use and future research can be drawn from the results of this study. Clinically, results provide support for significant and meaningful relationships between physical activity and self-efficacy for physical activity. The differences in adjusted mean physical activity levels between individuals with high and low self-efficacy may yield important health benefits for adults with arthritis. As a factor associated with physical activity behavior in adults with arthritis, self-efficacy for physical activity may be a potential area of intervention to address the problem of inactivity. Programs designed to enhance self-efficacy for physical activity could help increase physical activity levels among adults with arthritis.

 From a research perspective, results of this study revealed similarities and differences in the factors associated with physical activity behavior between adults with arthritis and the general adult population. Given the differences, additional studies are recommended to examine the role of other potential determinants of physical activity among adults with arthritis, such as the type and location of arthritis, social support, and environmental barriers. The psychometric properties of the SEPA and OEE should be evaluated to determine the appropriateness of their use among adults with arthritis. Additionally, the appropriateness of using any of the scales (PASE, RASE, SEPA, and OEE) among minorities, individuals with low socioeconomic status, and individuals with low literacy should be evaluated. Further research is needed to determine factors associated with long-term, sustained, physical activity behavior in adults with arthritis.

## 6. Conclusion

This study was conducted to determine whether baseline levels of self-efficacy for physical activity, self-efficacy for arthritis self-management, and outcome expectations for exercise are associated with higher physical activity levels following the cessation of a structured exercise intervention for adults with arthritis. After controlling for baseline physical activity, neither self-efficacy for arthritis self-management nor outcome expectations for exercise related to 3-month physical activity levels, but the relationship between 3-month physical activity and self-efficacy for physical activity was clinically meaningful and statistically significant. The results highlight a potential area of intervention to address the problem of inactivity among adults with arthritis. Future research is needed to evaluate the ability of self-efficacy-enhancing programs to increase physical activity levels and to identify additional determinants of physical activity behavior in adults with arthritis.

## Figures and Tables

**Table 1:** Baseline demographic characteristics of participants (*n* = 130).

Characteristic	Mean (SD) or %
Age (*n* = 129)	71.5 (11.7)
Range	32.0–94.0
Gender (*n* = 127)	
Female	85.0
Race (*n* = 130)	
Caucasian	74.6
African American	20.0
American Indian	3.8
Other	1.6
Education (*n* = 129)	
>High school	58.9
Marital status (*n* = 127)	
Not married	54.3
Work situation (*n* = 130)	
Working full or part-time	10.0
Unemployed/disabled/LOA	16.1
Homemaker	15.4
Retired	58.5

*Note*. LOA: Leave of Absence.

**Table 2:** Baseline health-related characteristics of participants (*n* = 130).

Characteristic	Mean (SD) or %
SEPA (*n* = 128)	2.6 (0.85)
Range	1.0–4.4
OEE (*n* = 129)	4.1 (0.74)
Range	1.0–5.0
RASE (*n* = 126)	105.8 (12.6)
Range	63.0–140.0
Type of arthritis (*n* = 130)	
Osteoarthritis	54.6
Rheumatoid arthritis	24.6
Fibromyalgia	8.5
Duration of arthritis (years) (*n* = 119)	12.7 (12.1)
Range	1 month–62 years
Pain VAS (*n* = 124)	47.9 (25.7)
Range	0.0–100.0
Stiffness VAS (*n* = 126)	47.2 (30.6)
Range	0.0–100.0
Fatigue VAS (*n* = 127)	42.0 (27.0)
Range	0.0–100.0
HAQ (*n* = 130)	0.99 (0.62)
Range	0.0–2.5
Baseline PASE (*n* = 109)	92.8 (57.1)
Range	0.0–300.3
3-month PASE (*n* = 123)	73.6 (53.4)
Range	0.0–280.5

*Note*. PACE: People with Arthritis Can Exercise program. SEPA: Self-Efficacy for Physical Activity scale. OEE: Outcome Expectations for Exercise scale. RASE: Rheumatoid Arthritis Self-Efficacy scale. VAS: Visual Analog Scale. HAQ: Health Assessment Questionnaire. PASE: Physical Activity Scale for the Elderly.

**Table 3:** Adjusted** mean 3-month PASE by main explanatory variables.

Main explanatory variables	Adjusted mean 3-month PASE	95% confidence interval	*P *
RASE (*n* = 99)*			0.117
High^a ^	79.4	67.1, 91.6	
Low^b ^	65.5	53.1, 77.8	
OEE (*n* = 101)			0.326
High^c ^	77.3	63.9, 90.6	
Low^d ^	68.5	57.2, 79.8	
SEPA (*n* = 101)*			0.005
1	49.2	31.1, 67.4	
2	63.7	53.5, 74.0	
3	78.3	68.9, 87.6	
4	92.8	76.0, 109.5	

*P < 0.05.

**All three models adjusted for baseline physical activity as measured by the Physical Activity Scale for the Elderly (PASE). PASE = 92.68 in RASE model, PASE = 93.09 in OEE model. RASE: Rheumatoid Arthritis Self-Efficacy scale. OEE: Outcome Expectations for Exercise scale. SEPA: Self-Efficacy for Physical Activity scale.

^a^High RASE: >105.85. ^b^Low RASE: ≤105.85. ^c^High OEE: >4. ^d^Low OEE: ≤4.
